# Alcohol-Specific Mortality in People With Epilepsy: Cohort Studies in Two Independent Population-Based Datasets

**DOI:** 10.3389/fneur.2020.623139

**Published:** 2021-01-21

**Authors:** Hayley C. Gorton, Roger T. Webb, Rosa Parisi, Matthew J. Carr, Marcos DelPozo-Banos, Kieran J. Moriarty, W. Owen Pickrell, Ann John, Darren M. Ashcroft

**Affiliations:** ^1^School of Applied Sciences, University of Huddersfield, Huddersfield, United Kingdom; ^2^NIHR Greater Manchester Patient Safety Translational Research Centre, Manchester Academic Health Sciences Centre (MAHSC), University of Manchester, Manchester, United Kingdom; ^3^Division of Psychology & Mental Health, Centre for Mental Health and Safety, Manchester Academic Health Sciences Centre (MAHSC), Faculty of Biology, Medicine and Health, School of Health Sciences, University of Manchester, Manchester, United Kingdom; ^4^Division of Informatics, Imaging & Data Sciences, Manchester Academic Health Sciences Centre (MAHSC), Faculty of Biology, Medicine and Health, School of Health Sciences, University of Manchester, Manchester, United Kingdom; ^5^Centre for Pharmacoepidemiology and Drug Safety, Manchester Academic Health Sciences Centre (MAHSC), Faculty of Biology, Medicine and Health, School of Health Sciences, University of Manchester, Manchester, United Kingdom; ^6^Farr Institute, Swansea University Medical School, Swansea, United Kingdom; ^7^Bolton NHS Foundation Trust, Bolton, United Kingdom; ^8^Neurology and Molecular Neuroscience Research Group, Swansea University Medical School, Swansea University, Swansea, United Kingdom; ^9^Neurology Department, Morriston Hospital, Swansea Bay University Health Board, Swansea, United Kingdom

**Keywords:** epilepsy, alcohol, alcohol-specific, cohort, observational

## Abstract

**Objectives:** The risk of dying by alcohol-specific causes in people with epilepsy has seldom been reported from population-based studies. We aimed to estimate the relative risk of alcohol-specific mortality in people with epilepsy, and the extent to which problematic alcohol use was previously identified in the patients' medical records.

**Method:** We delineated cohort studies in two population-based datasets, the Clinical Practice Research Datalink (CPRD GOLD) in England (January 01, 2001–December 31, 2014) and the Secure Anonymised Information Linkage (SAIL) Databank in Wales (January 01, 2001–December 31, 2014), linked to hospitalization and mortality records. People with epilepsy were matched to up to 20 persons without epilepsy on gender, age (±2 years) and registered general practice. We identified alcohol-specific death from Office for National Statistics (ONS) records using specified ICD-10 codes. We further identified prescriptions, interventions and hospitalisations related to alcohol use.

**Results:** In the CPRD GOLD, we identified 9,871 individuals in the incident epilepsy cohort and 185,800 in the comparison cohort and, in the SAIL Databank, these numbers were 5,569 and 110,021, respectively. We identified a five-fold increased risk of alcohol-specific mortality in people with epilepsy vs. those without the condition in our pooled estimate across the two datasets (deprivation-adjusted HR 4.85, 95%CI 3.46–6.79).

**Conclusions:** People with epilepsy are at increased risk of dying by an alcohol-specific cause than those without the disorder. It is plausible that serious alcohol misuse could either contribute to the development of epilepsy or it could commence subsequent to epilepsy being diagnosed. Regardless of the direction of the association, it is important that the risk of dying as a consequence of alcohol misuse is accurately quantified in people affected by epilepsy. Systematically-applied, sensitive assessment of alcohol consumption by healthcare professionals, at opportunistic, clinical contacts, with rapid access to quality treatment services, should be mandatory and play a key role in reduction of health harms and mortality.

## Introduction

People with epilepsy are known to die prematurely compared to the rest of the general population (1, 2). However, the need for improved identification of specific causes of death, and associated risks of these deaths in people with epilepsy has been recognized. Devinsky et al. suggest that the contribution of alcohol to death in people with epilepsy may be underestimated (2). Recently, we reported that people with epilepsy were three-times more likely to die from unnatural causes than those without (3). Whilst we did not study alcohol-specific mortality, we noted the greater frequency of alcohol misuse in people with epilepsy (6.0%) vs. the comparison cohort (1.4%). This warranted further investigation.

Death due to alcohol-dependence syndrome in people with epilepsy was associated with a standardized mortality rate of 3.9 (95% CI 1.8–7.4) in a prevalent epilepsy cohort identified from attendees at an Austrian epilepsy hospital clinic (4). In 2016, of the 137,000 epilepsy deaths and 14.7 million epilepsy Disability-adjusted life years (DALYs) in the United Kingdom, an estimated 17,000 epilepsy deaths and 1.5 million epilepsy DALYs were attributed to alcohol, representing 12.7% of all deaths and 10.2% of all DALYs due to epilepsy (5). Alcohol-related death is a wide definition that may include end-organ disease attributed to alcohol (6). To our knowledge, the only published study that specifically examines alcohol-related death in people with epilepsy is a Finnish register-based cohort in which people with epilepsy were compared to a population-based reference cohort. A three-fold elevation in risk of death due to alcohol-related diseases and accidental poisoning by alcohol was observed (HR 3.38, 95%CI 2.76–4.16) (7). These deaths were identified by certain codes from the *International Classification of Diseases and Related Health Problems, ninth and tenth editions (ICD-9, ICD-10)*. The specific ICD-10 codes used have some parallels with, but are not identical to those which the Office for National Statistics (ONS) in the UK identified as alcohol-related deaths in the UK until 2016 in official death statistics (6). In 2017, the ONS replaced the definition for alcohol-related death with one for alcohol-specific death, in response to an expert consultation. To our knowledge, this is the first study to utilize this new categorization to examine the risk of alcohol-specific death in people with epilepsy.

The aims of this study were therefore to (i) estimate the incidence and relative risk of alcohol-specific death in people with epilepsy vs. persons without the condition; and (ii) estimate the extent of alcohol-related consultation, treatment or referral in relation to epilepsy status and alcohol-related death.

## Materials and Methods

### Data Source

We examined the Clinical Practice Research Datalink (CPRD GOLD), an electronic healthcare dataset which comprises general practice data from 7% of the UK population (8). It includes demographic, diagnostic, prescription, referral and test data from individuals in registered practices; and is considered to be broadly representative of the UK population as a whole in terms of its age, gender and ethnicity distributions. We defined our study populations from a subset of practices that were linked to Hospital Episode Statistics (HES) and Office for National Statistics (ONS) data; all of which were in England and represented ~58% of all practices in the CPRD. The HES provides hospital discharge dates and diagnoses whilst ONS provides date and causes of death (8). We extracted the incident epilepsy and matched cohorts from the July 2015 release of the CPRD which included 7,378,852 patients from 387 practices in England with routine data linkage implemented. We also estimated the incidence rates and relative risks of alcohol-specific death in the Secure Anonymised Information Linkage (SAIL) Databank, which enabled meta-analysis of relative risk estimates across two independent datasets. The SAIL Databank is comprised of 13 health and social care datasets. These include the general practice dataset (GPD) which contains similar data to the CPRD and includes 76% of all general practices in Wales (practice *n* = 360, patients *n* = 4,052,388) (9). ONS death data is detailed within the Annual District Death Extract (ADDE) and hospital discharge dates and diagnoses are included in the Patient Episode Database for Wales (PEDW) (10). As the CPRD cohort only included practices in England, there was no overlap with those individuals in the SAIL Databank. Approvals to conduct these studies in the CPRD and the SAIL Databank were granted by the Independent Scientific Advisory Committee (ISAC) of the Medicine and Healthcare Regulatory Agency (MHRA) (protocol 15_046RA2R) and the Information Governance Review Panel (approval 0728), respectively.

### Delineation of the Matched-Cohort

Epilepsy diagnoses were identified from the general practice data in the CPRD and the SAIL Databank using Read codes, version 2 (11). Separately in each dataset, we identified people as having incident epilepsy if they had the first evidence of a code for epilepsy diagnosis and an associated prescription for an antiepileptic drug (AED) in the month before or 6 months after the diagnosis. The requirement for both an epilepsy diagnosis and AED treatment is suggested to increase the reliability of the ascertainment of epilepsy in observational studies, by the International League Against Epilepsy (ILAE) (12) and is a definition we have previously applied in epidemiological studies of epilepsy (13). The use of primary care records in the SAIL Databank to identify people with epilepsy has been validated (14). The codes used to identify epilepsy diagnoses and AEDs, and an explanation of the verification process, are available on www.clinicalcodes.org (15). A minimum age of 18 years on study entry was imposed to align with the minimum legal age of alcohol purchase in the UK. Individuals were also required to have at least 1 year of prior registration in the practice, during which time they could not have had a diagnosis of epilepsy. This increased our confidence that we had delineated an incident epilepsy cohort. We defined the study period as January 1, 2001–March 31, 2014 in the CPRD and January 1, 2001–December 31, 2014 in the SAIL Databank, to correspond with linkage availability.

We matched each individual with epilepsy to up to 20 individuals without an epilepsy diagnosis on year of birth (±2 years), gender and general practice. Individuals were eligible for inclusion in the comparison cohort if they had at least 1 year of prior registration in the general practice and were alive on the index epilepsy date of the matched individual with epilepsy, and follow-up began on this date. Individuals were followed until the earliest date of: death, patient left practice, end of data collection in the practice, or the final observation date for the study cohort.

### Identification of Alcohol-Specific Mortality

We used the ONS definition of alcohol-specific mortality, which was introduced in 2017 to replace the previous definition of alcohol-related mortality, following a UK government consultation with leading experts in the field (6). It includes underlying cause of death codes assigned from the ICD-10 (Table 1).

**Table 1:** Reproduced from Office for National Statistics.

**ICD-10 Code**	**Condition**
E24.4	Alcohol-induced pseudo-Cushing's syndrome
F10	Mental and behavioral disorders due to use of alcohol
G31.2	Degeneration of nervous system due to alcohol
G62.1	Alcoholic polyneuropathy
G72.1	Alcoholic myopath
I42.6	Alcoholic cardiomyopathy
K29.2	Alcoholic gastritis
K70	Alcoholic liver disease
K85.2	Alcohol-induced acute pancreatitis
K86.0	Alcohol induced chronic pancreatitis
Q86.0	Fetal alcohol syndrome (dysmorphic)
R78.0	Excess alcohol blood levels
X45	Accidental poisoning by and exposure to alcohol
X65	Intentional self-poisoning by and exposure to alcohol
Y15	Poisoning by and exposure to alcohol, undetermined intent

*Alcohol-specific deaths in the UK: registered in 2016. Newport: Office for National Statistics; 2016*.

### Statistical Analysis

We used descriptive statistics to report baseline characteristics of age, gender and area-level deprivation. We estimated alcohol-specific mortality rate per 10,000 person-years and the risk of alcohol-specific mortality as a hazard ratio (HR), using stratified Cox-proportional hazards models to account for the matched cohort design. We report estimates separately based on the CPRD and the SAIL Databank data, and meta-analyzed estimates using the DerSimonian-Laird Random Effects model (16). To better understand whether alcohol consumption had been acknowledged in primary or secondary care records prior to death in those who died by alcohol-specific causes, we estimated the proportion of individuals who were: (i) coded as a heavy drinker; (ii) received a prescription for acamprosate, disulfiram, nalmefene or naltrexone for alcohol dependence; (iii) referred for psychological support for alcohol use and (iv) hospital admission for alcohol-related causes; in relation to their epilepsy status. We used codes and algorithms that were previously published to define these variables (17), and they are available on www.clinicalcodes.org.uk (15). Alcohol consumption was defined in relation to the national guidance on intake, which was in place during the study's observation period (18).

## Results

### Baseline Characteristics

We matched 9,871 people with incident epilepsy with 185,800 individuals in the comparison cohort in the CPRD (Table 2). The median age on entry was 57 (IQR 39–73) in the epilepsy cohort and 58 (IQR 40–74) in the comparison cohort and 51% of both cohorts were male. The epilepsy cohort was followed-up for a median of 3.2 years (1.2–6.6) whilst the comparison cohort was followed-up for a median of 4.3 years (1.9–7.7). 9.7% of people in the epilepsy cohort and 6.4% in the comparison cohort were categorized as being a “heavy drinker.” Death due to any cause was a more common reason for end of follow-up in the epilepsy cohort (23.1%) than in the comparison cohort (11%). The distribution of covariates was similar in the analysis conducted in the SAIL Databank, which included 5,569 people with incident epilepsy and 110,021 people in the comparison cohort (Table 2).

**Table 2:** Baseline characteristics of epilepsy and comparison cohorts in the CPRD and SAIL databank.

	**CPRD**		**SAIL**	
**Baseline characteristic**	**Epilepsy cohort** **(*n* = 9,871)**	**Comparison cohort** **(*n* = 185,800)**	**Epilepsy cohort** **(*n* = 5,569)**	**Comparison cohort** **(*n* = 110,021)**
Male	5,062 (51·3%)	95,647 (51.5%)	2,874 (51.6%)	56,729 (51.6%)
Median age on entry (IQR)	57 (39–73)	58 (40–74)	54 (37–70)	54 (37–70)
**Level of deprivation^a^**				
1 (least deprived)	1,908 (19.3%)	40,105 (21.5%)	905 (16.3%)	21,022 (19.1%)
2	2,190 (22.2%)	43,077 (23.2%)	928 (16.7%)	20,186 (18.4%)
3	1,870 (18.9%)	37,066 (20.0%)	1,248 (22.4%)	24,158 (22.0%)
4	1,959 (19.9%)	35,257 (19.0%)	1,204 (21.6%)	22,714 (20.7%)
5 (most deprived)	1,937 (19.6%)	30,111 (16.2%)	1,280 (23.0%)	21,776 (19.8%)
missing	7 (0.1%)	184 (0.1%)	<5 (0.1%)	165 (0.2%)
**Follow up**				
Median follow-up time (years)	3.2 (1.2–6.6)	4.3 (1.9–7.7)	4.7 (IQR 1.8–8.6)	5.9 (IQR 2.6–9.7)
**Alcohol consumption^b^^,^^c^**				
Non-drinker	1,774 (18.0%)	26,360 (14.2%)		
Former drinker	315 (3.2%)	2,772 (1.5%)		
Occasional drinker	1,248 (12.6%)	24,087 (13.0%)		
Moderate drinker	3,359 (34.0%)	74,782 (40.3%)		
Heavy drinker	958 (9.7%)	11,881 (6.4%)		
Missing	2,217 (22.5%)	45,968 (24.6%)		

*Data on alcohol consumption could only be calculated in the CPRD*.

a *Based-on quintiles of multiple deprivation where 1 is the most deprived and 5 is the least deprived*.

b *Alcohol consumption status estimated at index date based on algorithm by Parisi et al. (17)*.

c *P < 0.001. The black shaded portion represents that the descriptive analysis was not conducted in the SAIL Databank*.

### Risk of Alcohol-Specific Mortality in People With Epilepsy

There were 29 alcohol-specific deaths in the incident epilepsy cohort and 106 in the comparison cohort in the CPRD, and the corresponding numbers were 18 and 96 in the SAIL Databank, respectively. The deprivation-adjusted hazard ratios for alcohol-specific mortality in people with epilepsy vs. those without were 5.47 (95%CI 3.52–8.48) in the CPRD and 4.08 (95%CI 2.42–6.90) in the SAIL Databank, and the pooled estimate was 4.85 (95%CI 3.46–6.79) (Table 3).

**Table 3:** Incidence and relative-risk of Alcohol-specific death in people with incident epilepsy vs. comparison cohort.

**Alcohol-related death**	**Number**	**Rate/10,000PY** **(95%CI)**	**Number**	**Rate/10,000 PY** **(95%CI)**	**HR (95% CI)**	**Deprivation-adjusted HR** **(95%CI)**
CPRD	Epilepsy cohort (*n* = 9,871; PY = 41,253)		Comparison cohort (*n* = 185,800; PY = 916,572)			
	29	7.0 (4.7–10.1)	106	1.2 (0.9–1.4)	6.09 (3.96–9.37)	5.47 (3.52–8.48)
SAIL	Epilepsy cohort (*n* = 5,569; PY = 30,011)		Comparison cohort (*n* = 110,021; PY = 685,575)			
	18	6.0 (3.6–9.5)	96	1.4 (1.1–1.7)	4.40 (2.62–7.37)	4.08 (2.42–6.90)
**Meta-analyzed hazard ratios**					5.33 (3.83–7.42)	4.85 (3.46–6.79)

### Alcohol Consumption and Alcohol-Related Diagnoses, Treatment, Psychological Support and Hospitalization

Amongst those in the CPRD who died due to alcohol specific causes, compared to the matched cohort, a greater proportion of people with epilepsy had medication prescribed for alcohol dependence (24.1 vs. 9.3%; *p* = 0.03). There were proportionally more people in the epilepsy cohort than the comparison cohort who were referred for psychological support for alcohol use, but this was not statistically significant (20.7 vs. 11.3%; *P* = 0.19). There was no statistically significant difference between coding as a “heavy drinker” at any time prior to death in the epilepsy and comparison cohorts in the CPRD (79.3 vs. 67.0%; *p* = 0.2). A similar proportion of each group had ever been hospitalized for reasons related to alcohol use (84.9 vs. 86.2%). A greater proportion of the epilepsy cohort than the comparison cohort had a history of alcohol misuse, as determined by any of these indicators, prior to index date (i.e., prior to epilepsy diagnosis), but this difference was not statistically significant (79.3 vs. 72.6%; *p* = 0.47).

## Discussion

### Elevated Risk of Alcohol-Specific Death

We have shown across two, large population-based cohort studies that people with epilepsy are approximately five times more likely to die from alcohol-specific causes than persons without the condition (deprivation-adjusted meta-analyzed HR: 4.85, 95%CI 3.46–6.79). Of those people who died from alcohol-specific causes, the majority had a hospital admission that was attributed to alcoholism (epilepsy cohort: 86.2%; comparison cohort: 84.9%). Many were coded as being a heavy drinker in primary care records, but fewer were prescribed medication for alcohol or received psychological support. Still, alcohol misuse had not been coded in primary care records prior to death in 20 to 28% of people. For those individuals whose alcohol misuse has been identified in primary care or due to previous hospitalization, thought should be given as to how to maximize these contact opportunities to try to prevent the sequel of events that culminate in hospitalization and premature death.

Our estimate of relative risk of dying by an alcohol-specific cause in people with epilepsy is of a greater magnitude than that predicted by Nevalainen et al. (HR 4.85, 95%CI 3.46–6.79 compared to 3.44, 95%CI 3.11–3.71) from Finnish mortality records. That study's somewhat lower estimate could be a reflection in differences in habits in population drinking habits or access to healthcare in Finland, England and Wales. Estimates of per capita alcohol consumption in 2016 were higher in the UK (11.4 L/capita) than Finland (10.7 L/capita) (19). It may also be a function of study design. We used a more recent time period than the 2008 study-end in the Finnish study. We both used ICD-10 codes to define the outcome, but the Finnish study reported alcohol-related deaths whereas we report alcohol-specific deaths. In some aspects we were broader; for example inclusion of intentional poisoning with alcohol, whereas the Finnish investigators included only accidental poisoning (7). In other aspects, the previous study employed broader codes, for example, “Special epilepsy syndromes” of which alcohol is one potential cause. In a systematic review of systematic reviews, alcohol involvement was identified as a high risk cause of alcohol-related mortality (20).

Other studies have reported the proportion or likelihood of alcohol involvement in death among persons with epilepsy. The National Drug-Related Deaths Index of Ireland is a surveillance system that records all causes of death in people who are dependent on alcohol or drugs, or die due to poisoning (21). Two percent of individuals in this register were known to have epilepsy and 81% of these had a history of alcohol dependence prior to death. In a nested case-control study conducted in the General Practice Research Database (the predecessor to the CPRD), Risdale et al. (22) identified that amongst people with epilepsy, those who died during follow-up were more likely to have a record of alcohol misuse (OR 2.96, 95%CI 2.25–3.89) than those who did not die. Our study design does not permit us to compare to these estimates. It does, however highlight that alcohol misuse have been identified as a major contributor to premature death in people with epilepsy. We have not found any literature that maps primary care recording of alcohol misuse prior to alcohol-specific death in people with epilepsy.

In the UK General Practice Survey, there was a three-fold increased risk of death (HR 2.9, 95%CI 1.5–5.7) if alcohol was a cause of epilepsy compared to if it was not (23). In a meta-analysis of studies, a relative risk of 2.19 (95%CI 1.83–2.63) was estimated for epilepsy or unprovoked seizures in relation to alcohol consumption, and a dose-dependent relationship was evident (24). Consuming 12, 48, 72, and 96 g/day of alcohol was associated with relative risk increases, vs. non-drinkers, of 1.2 (95%CI; 1.1–1.2), 1.8 (95% CI 1.6–2.1), 2.4 (2.0, 3.0) and 3.3 (95%CI 2.5–4.3), respectively (25). One confounder in studies has been the difficulty in distinguishing between alcohol-withdrawal and non-withdrawal seizures.

A two-fold increased risk in Sudden Unexpected Death in Epilepsy (SUDEP) has been associated with alcohol dependence (26). This is not recorded in the datasets we used. Our findings must be interpreted with the important caveat that pre-existing alcohol dependence may have caused seizures or epilepsy in some cases. Alcohol has been suggested to be responsible for 1/20–1/5 of Disability-adjusted Life Years in people with epilepsy (27). The great majority of individuals in the epilepsy cohort (~80%) had codes for non-specific epilepsy. It was therefore not possible to determine whether seizures were classified as alcohol-induced. Our use of the incident epilepsy cohort somewhat overcomes this limitation by allowing us to identify the first epilepsy diagnosis and therefore understand whether any history of alcohol misuse or dependence was identified before or after this diagnosis. However, it is possible that alcohol dependence occurs some time before diagnosis, treatment or psychological intervention was recorded in the general practice data.

### Strengths and Limitations

To our knowledge, this is the first study to estimate the relative risk of death specific to alcohol misuse in those with epilepsy across two large population-based databases and to pool the estimates to optimize statistical precision. We have applied the most up-to-date definition of alcohol-specific death that is now being used in routine mortality registration in England & Wales (6). This will enable any recommendations that we make to be implemented and measured against this standard definition in future years. We report for the first time estimates for primary care identification of alcohol misuse or dependence prior to alcohol-specific death in a matched epilepsy and comparison cohort.

We could not stratify by type of epilepsy or seizure frequency due to limitations in recording, nor could we report relative risk for each individual alcohol-specific cause. This is due to the low numbers of death at this more granular analytical level, which could not be reported without compromising anonymity, and that would produce imprecise relative risk estimates. The availability of self-reported alcohol consumption data in the CPRD is likely to be incomplete, and may not be entirely accurate, influenced by the individual's willingness to accurately report alcohol use and opportunity to record this information, such as during primary care consultations. Given that alcohol misuse and dependence can induce seizures, whether the primary cause of epilepsy or not, it might be expected that people with epilepsy would have their alcohol status recorded more so than those people in our comparison cohort. This could artificially widen the gap in reporting. There should be no differential misclassification between the groups for prescription or psychological referral recording.

### Implications for Clinicians

Regardless of the direction of the association between epilepsy and alcohol misuse, clinicians should be mindful of the large elevation in risk of alcohol-specific death among people with epilepsy. Any progress toward stopping drinking alcohol can have a beneficial effect on survival of people with alcohol-related cirrhosis (28). Given the magnitude of the risk elevation observed, it is important that clinicians who are treating people with epilepsy are aware of alcohol misuse. This might enable targeted intervention and person-centered holistic care, both of which have been recommended to enhance the care for people with alcohol-related diseases (29). Indeed, exploration of alcohol use in people with epilepsy is recommended through use of the “SUDEP and seizure safety” checklist in the UK (30). Additional, candid discussions about level of alcohol use at the point of epilepsy diagnosis and at regular reviews could be warranted. It should be noted that, in the same study cohort, the incidence of alcohol-specific death is lower than we previously observed for other potentially preventable causes of mortality, such as accident and suicide (3). However, the considerable morbidity related to alcohol-induced seizures in epilepsy, whether this was the initial cause or not, supports the need to discuss the impact of alcohol misuse among epilepsy. Indeed, the observed difference in rate might reflect the immediacy that may be related to accident or suicide, whereas many of the alcohol-specific causes are a culmination over time.

The historical, global stigmatization of people with epilepsy (31) or alcohol problems, of particular concern when the two co-exist, can lead to denied or delayed acceptance, presentation, detection, treatment and health risks, including death, from the two conditions. The commonality of concurrent mental illness in people with epilepsy and the independent increased risk of accident (32), suicide (33), and alcohol use related to mental illness should not be ignored. Upstream identification, treatment and management of mental illness in people with epilepsy has the potential to mitigate preventable causes of death, a priority described in the 2016 Call to Action on this issue (2).

## Conclusion

We have observed a five-fold elevated risk of alcohol-specific deaths in people with epilepsy compared to those without. Many but not all of those who died had a hospitalization or primary care event signifying excessive alcohol use. Hence, in people with epilepsy, systematically-applied, sensitive assessment of alcohol consumption (34) by healthcare professionals, at opportunistic, clinical contacts, with rapid access to quality treatment services, should be mandatory and play a key role in reduction of health harms and mortality.

## Data Availability Statement

The data analyzed in this study are subject to the following licenses/restrictions: The clinical codes used in this study are published on Clinicalcodes.org. Electronic health records are, by definition, considered “sensitive” data in the UK by the Data Protection Act and cannot be shared *via* public deposition because of information governance restriction in place to protect patient confidentiality. Access to data are available only once approval has been obtained through the individual constituent entities controlling access to the data. The primary care data can be requested *via* application to the Clinical Practice Research Datalink (www.cprd.com) or the SAIL Databank (https://www.saildatabank.com/application-process). Requests to access these datasets should be directed to enquiries@cprd.com.

## Ethics Statement

The studies involving human participants were reviewed and approved by ISAC of the CPRD, MHRA, and IGRP of the SAIL Databank. Written informed consent for participation was not required for this study in accordance with the national legislation and the institutional requirements.

## Author Contributions

HCG, RTW, and DMA contributed to the conceptualization of the study. HCG, RTW, DMA, AJ, MD-B, and MJC designed the study. HCG, MJC, and MD-B extracted data. HCG conducted the statistical analysis. HCG, DMA, RTW, MJC, MD-B, AJ, and KJM contributed to interpretation of results. RP developed the algorithm to quantify alcohol consumption. HCG drafted the initial manuscript. KJM contributed to the section on alcohol screening, misuse, and treatment services. RTW, DMA, RP, MJC, KJM, WOP, AJ, and MD-B amended and revised the manuscript. All authors approved the submitted version.

## Conflict of Interest

HCG is a member of Epilepsy Action Health & Clinical Advisory Panel. This is a voluntary position. The remaining authors declare that the research was conducted in the absence of any commercial or financial relationships that could be construed as a potential conflict of interest.

## References

[1] Thurman DJ Logroscino G Beghi E Hauser A Hesdorffer DC Newton CR. The burden of premature mortality of epilepsy in high-income countries: a systematic review from the Mortality Task Force of the International League Against Epilepsy. Epilepsia. (2017) 58:17–26. 10.1111/epi.13604 27888514 PMC7004822

[2] Devinsky O Spruill T Thurman D Friedman D. Recognizing and preventing epilepsy-related mortality. A call for action. Neurol. (2016) 86:779–86. 10.1212/WNL.0000000000002253 26674330 PMC4763802

[3] Gorton HC Webb RT Carr MJ DelPozo-Banos M John A Ashcroft DM. Risk of unnatural mortality in people with epilepsy. JAMA Neurology. (2018) 75:929–38. 10.1001/jamaneurol.2018.0333 29630689 PMC6075702

[4] Granbichler CA Oberaigner W Kuchukhidze G Bauer G Ndayisaba J-P Seppi K. Cause-specific mortality in adult epilepsy patients from Tyrol, Austria: hospital-based study. J Neurol. (2015) 262:126–33. 10.1007/s00415-014-7536-z 25344746 PMC4289978

[5] World Health Organization (2018). Available online at: https://www.who.int/publications/i/item/9789241565639 (accessed September 12, 2020).

[6] Office for National Statistics (2017). Available online at: https://www.ons.gov.uk/peoplepopulationandcommunity/birthsdeathsandmarriages/deaths/articles/theimpactofusingthenewdefinitionofalcoholspecificdeaths/2017-10-27 (accessed September 12, 2020).

[7] Nevalainen O Raitanen J Ansakorpi H Artama M Isojarvi J Auvinen A. Long-term mortality risk by cause of death in newly diagnosed patients with epilepsy in Finland: a nationwide register-based study. Eur J Epidemiol. (2013) 28:981–90. 10.1007/s10654-013-9848-1 24072507

[8] Herrett EG ABhaskaran M Forbes K Mathur H van Staa R Smeeth TL. Data resource profile: clinical practice research datalink (CPRD). Int J Epidemiol. (2015) 44:1740. 10.1093/ije/dyv098 26050254 PMC4521131

[9] SAIL Databank (2020). Available online at: https://saildatabank.com (accessed August 10, 2020).

[10] John AD Kosnes M Gunnell L Scourfield D Ford J Lloyd DV. Suicide Information Database-Cymru: a protocol for a population-based, routinely collected data linkage study to explore risks and patterns of healthcare contact prior to suicide to identify opportunities for intervention. BMJ Open. (2014) 4:e006780. 10.1136/bmjopen-2014-006780 25424996 PMC4248097

[11] Chisholm J. The read clinical classification. BMJ. (1990) 300:1092. 10.1136/bmj.300.6732.1092 2344534 PMC1662793

[12] Thurman DJ Beghi E Begley CE Berg AT Buchhalter JR Ding D. Standards for epidemiologic studies and surveillance of epilepsy. Epilepsia. (2011) 52(Suppl 7):2–26. 10.1111/j.1528-1167.2011.03121.x 21899536

[13] Gorton HC Webb RT Pickrell WO Carr MJ Ashcroft DM. Risk factors for self-harm in people with epilepsy. J Neurol. (2018) 265:3009–16. 10.1007/s00415-018-9094-2 30357466 PMC6244650

[14] Fonferko-Shadrach B Lacey AS White CP Powell HR Sawhney IM Lyons RA. Validating epilepsy diagnoses in routinely collected data. Seizure. (2017) 52:195–8. 10.1016/j.seizure.2017.10.008 29059611 PMC5703030

[15] Springate DA Kontopantelis E Ashcroft DM Olier I Parisi R Chamapiwa E. ClinicalCodes: an online repository to improve the validity and reproducibility of research using electronic medical records. PLoS One. (2014) 9:e99825. 10.1371/journal.pone.0099825 24941260 PMC4062485

[16] Higgins JP Green S editors. Cochrane Handbook for Systematic Reviews of Interventions. Chichester: The Cochrane Collaboration (2011).

[17] Parisi R Webb RT Carr MJ Moriarty KJ Kleyn E Griffiths CE. Alcohol-related mortality in patients with psoriasis. A population-based cohort study. JAMA Dermatol. (2017) 153:1256–62. 10.1001/jamadermatol.2017.3225 28914955 PMC5817445

[18] Department of Health Sensible Drinking The Report of an Inter-Departmental Working Group. London: Department of Health (1995).

[19] Ritchie H Roser M Available online at: https://ourworldindata.org/alcohol-consumption (2018). (accessed August 10, 2020).

[20] Mbizvo GK Bennett K Simpson CR Duncan SE Chin RFM. Epilepsy-related and other causes of mortality in people with epilepsy: A systematic review of systematic reviews. Epilepsy Res. (2019) 157:106192. 10.1016/j.eplepsyres.2019.106192 31526975

[21] Lynn E Lyons S Langan Y Craig S Doherty C. The role of alcohol dependency in deaths among people with epilepsy recorded by the National Drug-Related Deaths Index (NDRDI) in Ireland, 2004–2013. Seizure. (2017) 45:52–5. 10.1016/j.seizure.2016.11.014 27919010

[22] Risdale L Charlton J Ashworth M Richardson MP Gulliford MC. Epilepsy mortality and risk factors for death in epilepsy: a population-based study. Br J Gen Pract. (2011) 61:e271–78. 10.3399/bjgp11X572463 21619751 PMC3080232

[23] Lhatoo SD Johnson AL Goodridge DM MacDonald BK Sander WAS Shorvon SD. Mortality in epilepsy in the first 11 to 14 years after diagnosis: multivariate analysis of a long-term, prospective, population-based cohort. Ann Neurol. (2001) 49:336–44. 10.1002/ana.70 11261508

[24] Samokhvalov AV Irving H Mohapatra S Rehm J. Alcohol consumption, unprovoked seizures, and epilepsy: a systematic review and meta-analysis. Epilepsia. (2010) 51:1177–84. 10.1111/j.1528-1167.2009.02426.x 20074233

[25] Public Health England (2018). Available online at: https://assets.publishing.service.gov.uk/government/uploads/system/uploads/attachment_data/file/733108/alcohol_public_health_burden_evidence_review_update_2018.pdf (accessed December 4, 2020).

[26] Sveinsson O Andersson T Mattsson P Carlsson S Tomson T. Clinical risk factors in SUDEP. Neurology. (2020) 94:e419–29. 10.1212/wnl.0000000000008741 31831600 PMC7079690

[27] Beghi E Giussani G Nichols E Abd-Allah F Abdela J A. Abdelalim Global, regional, and national burden of epilepsy, 1990–2016: a systematic analysis for the Global Burden of Disease Study 2016. Lancet Neurol. (2019) 18:357–75. 10.1016/S1474-4422(18)30454-X 30773428 PMC6416168

[28] Verrill C Markham H Templeton A Carr NJ Sheron N. Alcohol-related cirrhosis-early abstinence is a key factor in prognosis, even in the most severe cases. Addiction. (2009) 104:768–74. 10.1111/j.1360-0443.2009.02521.x 19344445

[29] Moriarty KJ. Alcohol care teams: where are we now? Frontline Gastroenterol. (2020) 11:293–302. 10.1136/flgastro-2019-101241 32582422 PMC7307041

[30] Shankar R Newman C McLean B Anderson T Obe JH. Can technology help reduce risk of harm in patients with epilepsy? Br J Gen Pract. (2015) 65:448–9. 10.3399/bjgp15X686413 26324472 PMC4540375

[31] de Boer H.M. Epilepsy stigma: Moving from a global problem to global solutions. Seizure. (2010) 19:630–6. 10.1016/j.seizure.2010.10.017 21075013

[32] Crump C Sundquist K Winkleby MA Sundquist J. Mental disorders and risk of accidental death. BJ Psych. (2013) 203:297–302. 10.1192/bjp.bp.112.123992 23969485

[33] Singhal A Ross J Seminog O Hawton K Goldacre MJ. Risk of self-harm and suicide in people with specific psychiatric and physical disorders: comparisons between disorders using English national record linkage. J R Soc Med. (2014) 107:194–204. 10.1177/0141076814522033 24526464 PMC4023515

[34] Saunders JB Aasland OG Babor TF De La Fuente JR Grant M. Development of the alcohol use disorders identification test (AUDIT): WHO collaborative project on early detection of persons with harmful alcohol consumption-II. Addiction. (1993) 88:791–804. 8329970 10.1111/j.1360-0443.1993.tb02093.x

